# Studying complex interventions: reflections from the FEMHealth project on evaluating fee exemption policies in West Africa and Morocco

**DOI:** 10.1186/1472-6963-13-469

**Published:** 2013-11-08

**Authors:** Bruno Marchal, Sara Van Belle, Vincent De Brouwere, Sophie Witter

**Affiliations:** 1 Department of Public Health, Institute of Tropical Medicine, Antwerp, Nationalestraat 155, Antwerpen B-2000, Belgium; 2 Faculty of Public Health and Policy, Department of Global Health Development, London School of Hygiene and Tropical Medicine, London, UK; 3 Department of Public Health, Tropical Medicine, Institute of Tropical Medicine, Antwerp, Belgium; 4 Immpact, Institute of Applied Health Studies, University of Aberdeen, Aberdeen, UK

**Keywords:** Fee exemption policy, Policy implementation, Complex intervention, Research design, Complex adaptive systems, Theory-driven evaluation

## Abstract

**Background:**

The importance of complexity in health care policy-making and interventions, as well as research and evaluation is now widely acknowledged, but conceptual confusion reigns and few applications of complexity concepts in research design have been published. Taking user fee exemption policies as an entry point, we explore the methodological consequences of 'complexity’ for health policy research and evaluation. We first discuss the difference between simple, complicated and complex and introduce key concepts of complex adaptive systems theory. We then apply these to fee exemption policies.

**Design:**

We describe how the FEMHealth research project attempts to address the challenges of complexity in its evaluation of fee exemption policies for maternal care. We present how the development of a programme theory for fee exemption policies was used to structure the overall design. This allowed for structured discussions on the hypotheses held by the researchers and helped to structure, integrate and monitor the sub-studies. We then show how the choice of data collection methods and tools for each sub-study was informed by the overall design.

**Discussion:**

Applying key concepts from complexity theory proved useful in broadening our view on fee exemption policies and in developing the overall research design. However, we encountered a number of challenges, including maintaining adaptiveness of the design during the evaluation, and ensuring cohesion in the disciplinary diversity of the research teams. Whether the programme theory can fulfil its claimed potential to help making sense of the findings is yet to be tested. Experience from other studies allows for some moderate optimism. However, the biggest challenge complexity throws at health system researchers may be to deal with the unknown unknowns and the consequence that complex issues can only be understood in retrospect. From a complexity theory point of view, only plausible explanations can be developed, not predictive theories. Yet here, theory-driven approaches may help.

## Background

User fee exemption for delivery and emergency obstetric care (EmOC) is a policy that has recently been introduced by a large number of countries, particularly in Africa, with the aim of enhancing access to care and improving maternal and neonatal outcomes [1,2]. The free cesarean section policy in Mali was introduced in 2005. It is applied nationally to all caesarean sections in the public sector, and in theory covers all facility-based costs (but not transport). In a three-way partition of costs, families are intended to fund the journey into the health centres, while communities fund the onward referral transport costs, and the state covers the costs of service provision, including accommodation, surgery, laboratory tests, and treatment of complications such as pre-eclampsia and ruptured uterus. Burkina Faso introduced a policy in 2006 that subsidised health facilities for 85% of the cost incurred for normal deliveries and caesarean sections. This policy followed several other programmes introduced by the Ministry of Health to improve care for pregnant women. In Morocco, the fee exemption policy initiated in 2008 was comprehensive, abolishing all user fees related to prenatal clinic consultations, normal deliveries, caesarean sections and all required drugs and consumables. It was part of a broad action plan for the health sector, which also included a programme to improve supply of drugs, a health workforce plan and interventions aimed at improving transfers of patients between health facilities. In Benin, the policy introduced in 2009 was more selective, covering caesarean sections only and reimbursing health facilities with a flat fee for each intervention carried out.

In 2011, the FEMHealth project was established, with EC funding, to conduct multi-disciplinary evaluations of fee exemption policies in these four countries. A scan of the literature shows that the number of studies or evaluations of such policies is rising [3-6]. These focus on policy effectiveness in terms of utilisation [7-10], equity [11] or cost-effectiveness [10]. Other focus on implementation issues [12-17] or barriers and facilitators [18]. Some studies focus on financing [19], or assess the effects of such policies on the health workforce [20,21] or health facilities [22]. Others still analyse the policy formulation process [23,24]. However, few studies of these studies are explicitly based on a hypothesis, a framework or a theory that would provide a basis for analysis or comparison of such policies. Exceptions include [13,25-30].

FEMHealth started from the perspective that these are complex policies, which therefore require tailored evaluation methodologies^a^. One of the objectives was to develop new methodological approaches for the evaluation of complex interventions in low-income countries. The importance of complexity for health care policy-making and interventions, as well as research and evaluation, is now acknowledged [31]. However, in the policy and health systems research (HPSR) literature, conceptual confusion is reflected by the interchangeable use of terms such as 'complicated’ and 'complex’ and divergent definitions of what makes a problem, an intervention or a specific setting complex. Similar problems affect discussions on what constitutes good designs for evaluation or research of complex interventions [32,33].

In this paper, we explore the consequences of the notion of complexity for health policy research and evaluation through the lens of the FEMHealth research programme. We first present a definition of complexity and key elements of complex systems theory, applying these to fee exemption policies. We then describe how the FEMHealth project attempted to address the complexity of such policies. We end with lessons learned in the process and some reflections on how to address complexity in health policy research and evaluation.

### What is complexity?

The notion of complexity has its origins in the field of natural sciences. Complexity theory absorbed elements of general systems theory, cybernetics, chaos theory and information theory. In all these fields, an evolution from reductionist Newtonian models of a well-ordered universe to paradigms that focus on non-linear dynamics started in the 1950s. Later, complex systems thinking was applied in management (see for instance [34,35]) and to the study of social phenomena by different social science disciplines [36,37], to development [38] and to policy analysis [39,40]. In health, there was a wave of attention at the beginning of the millennium, calling for use of complexity concepts in health [41-44], and some authors focused specifically on complexity in management of clinical care [42,45,46]. It took longer for complexity to surface in the mainstream of the public health literature. WHO, for instance, recently published a working paper on systems thinking and complexity in the frame of health system strengthening [47]. This late adoption may be due, in part, to the conceptual confusion regarding the definition of 'complexity’ and a fragmented application of complexity theory to health care [48,49].

We argue that fee exemption policies for maternal care are complex in two ways. First, they aim to address high maternal mortality - a typically complex problem involving a large number of social, cultural, economic, personal and systemic factors - and second, their implementation is complex.

The distinction between simple, complicated and complex problems made by Zimmerman and Glouberman [50] helps to make our point. These authors relate their definitions to causality and solutions:

•Simple problems have simple causes. Causality is linear and simple problems have standard solutions. These can be applied without specific expertise; technical skills are sufficient.

•Complicated problems consist of sets of simple problems, but cannot be reduced to them. They are compounded by scale and coordination problems. Solving complicated problems requires expertise and collaboration between experts. Formulae and instructions to solve complicated problems can be developed and are critical to success. If experts apply the formulae correctly, outcomes can be predicted.

•Complex problems include sets of simple and complicated problems to which they are not reducible. The interactions between determinants of the sub-problems can lead to non-linear causal relations between potential causes and outcomes. Also context-sensitivity can make a problem complex. As a consequence, outcomes are unpredictable. To solve complex problems, formulae and standardised solutions that proved effective in the past provide little guidance. Instead, complex problems are solved through fail-safe experiments that allow learning by doing or by making sense of events post facto.

Financial barriers to utilisation of health care services, and maternal mortality both fit Glouberman and Zimmerman’s definition of complex problems, determined as they are by multiple, interlinked factors. A fee exemption policy for pregnant women, that in essence consists of abolishing user fees for a certain group of the population, may seem at first a simple intervention: it can be introduced by mere administrative fiat, targets a well-specified group and has a simple causal chain: abolishing user fees reduces financial access barriers and leads thus to higher utilisation by pregnant women. This in turn is expected to contribute to more timely case management of complications of pregnancy or delivery and ultimately to lower morbidity and mortality. However, the actual implementation and uptake of the policy, and thus its effect, depends on the actors involved. They are likely to adapt the policy to the local context. The policy outcome will also be influenced by pre-existing context factors and determinants like poverty levels, health system coverage, quality of care, etc.

Jones presents a concise set of criteria than can help to decide when policy problems are likely to be complex [51]:

•the knowledge on cause-effect is limited (and thus predictability of outcomes is low)

•the consensus on policy issues and goals is limited (and thus divergence of actors’ goals is high)

•the required capacity to implement the policy is distributed (and thus requiring intensive communication and negotiation with many actors at all levels of the health system)

Jones’ three criteria for complexity of implementation are met in the case of fee exemption policies, inasmuch as there is limited high-quality evidence on the effects of fee exemption policies [52], the consensus amongst many of the actors in the health systems on the desirability of reforms is usually limited, and the policy relies for its success on the compliance of a large range of autonomous actors.

The complexity of fee exemption policies can also be assessed using the terminology of complex adaptive systems theory. This requires us first to consider what a 'system’ is. Morin defines a *system* as a unit made up by and organised through relations between elements (or agents), structures and actions (or processes) [53]. As with any system, *complex* systems consist of multiple elements, which interact with their environment, but some factors make them stand out: the nature of the interactions, the feedback loops, and the importance of the initial conditions and of the past. As a result, complex systems will display emergent behaviour and unpredictability. This applies as much to complex biological systems as to human social systems, including health systems [42,46].

To understand complex systems, one needs to understand the nature of the interactions between the elements. Typically, these interactions can be non-linear: small inputs may have large effects and vice versa. The effect of actions also depends on the initial conditions. In the case of a fee exemption policy, for instance, the result can be expected to be greater in regions with relatively high poverty levels compared to low poverty regions, assuming other barriers are similar.

In complex systems, positive and negative feedback loops contribute to emergent behaviour and unpredictability, and this is largely due to the human factor or the way human beings react to change. For instance, a policy that abolishes user fees may lead to higher utilisation of the hospital because it reduces financial barriers to access. This may lead to higher workloads for the health workers, and in response, health workers may impose new barriers to patient access in an effort to reduce stress. Other unintended effects may occur as overworked health workers become unfriendly to patients, leading to reduced patient satisfaction, which in turn may affect the decision to use the hospital’s services. Such feedback loops can often explain unexpected or perverse results. Furthermore, feedback loops may display time delays, in which case effects only become apparent after long periods of time. If managers or policymakers overreact in response to slow results of an intervention by initiating new interventions, the situation can change wildly (oscillation).

Complex systems are also path-dependent: outcomes of interventions are sensitive not only to initial (current) conditions, but also to decisions taken in the past. Applied to policymaking, this explains how present policy choices and implementation modes are determined by past choices. Managers used to raise organisational revenue by being paid fees for service by users, for example, find it hard to adjust to a fixed reimbursement per episode under exemption policies. This may explain why in Burkina Faso, for example, there has been a reversion to charging per item, contrary to the official fee subsidy policy [54].

Some of the above features already hint at the ability to 'self-organise’ that makes a complex system *adaptive*. Human agency is indeed the key factor that leads to adaptive change and evolution within complex systems. It also leads to variation in behaviour being the rule in complex adaptive systems rather than exceptional.

Applying the above concepts from complexity theory to user fee exemption policies, it could be argued that these policies offer an apparently simple solution (of changing the financing structure for specific priority services, thereby reducing financial barriers to utilisation) to the problem of high maternal mortality, a complex problem. The barriers to increasing service uptake are multiple, and changes to one factor are likely to lead to a ripple of reactions and feedback. Exemption policies rely on changing the behaviour of a wide range of actors, not least pregnant women and their households. Within the health system, multiple layers and organisations are involved. Furthermore, context and history play an important role, setting the scene and influencing the range of both policy and implementation options. The success of fee exemption policies is thus based on a large set of conditions or assumptions, which need to be made clear when developing a research design for policy analysis.

### Research designs to study a complex policy

One of FEMHealth’s objectives was to improve the knowledge base regarding the effectiveness, cost and impact of the removal of user fees for delivery care by carrying out comprehensive evaluations. The above discussion of complexity points to a number of consequences for the choice of research design and methods. Ideally, a study design for a complex intervention or problem should allow researchers to assess not only effectiveness but also the underlying processes so as to uncover the causal mechanisms. An understanding of how and in which context such policy can be expected to have similar impacts is central to its transferability. To do this, the study should explore the influence of key actors (including power analysis), and assess the organisational, social and historical context as well as the evolution of other policies that might have had an effect on the policy making process, implementation and observed outcomes. The design should deal with the significant time lags between policy decision, implementation and outcomes and the consequent risk of mismatch between research and policy time frames. Perhaps most challengingly, the design should be adaptive and allow for capturing the unexpected.

To deal with these challenges, FEMHealth adopted a multi-country, comparative case study design within a natural policy experiment perspective [55]. In principle, case studies allow for a holistic in-depth investigation of issues as they happen in their natural setting, whereby different sources of information and data collection methods can be used concurrently [56]. The case study design is in essence an adaptive design, as it facilitates exploration of a “*phenomenon within its real-life context, especially when the boundaries between phenomenon and context are not clearly evident*” [57].

In practice, we selected between 6 and 8 study sites in each country. However, a series of case studies of districts or facilities aimed at studying the implementation process would be insufficient. We also focused downstream and set out to assess quality of care and other outcomes at patient level. Furthermore, political sciences studies showed how the policy formulation and translation into a programme influence the actual implementation of a policy [58,59]. For this reason, the policy formulation process and the arrangements put in place by the central level were examined as well, alongside some investigation into the interaction with regional and international ideas and actors. We thus aimed at covering the multiple interactions between the spheres of communities and pregnant women, service providers, service managers, programme managers and policymakers. In practice, we combined assessments of the policy formulation, the implementation processes, provider and user perspectives, the intermediate outputs and the outcomes with qualitative and quantitative methods and tools.

### Using the programme theory as a structuring tool

**Figure 1:**
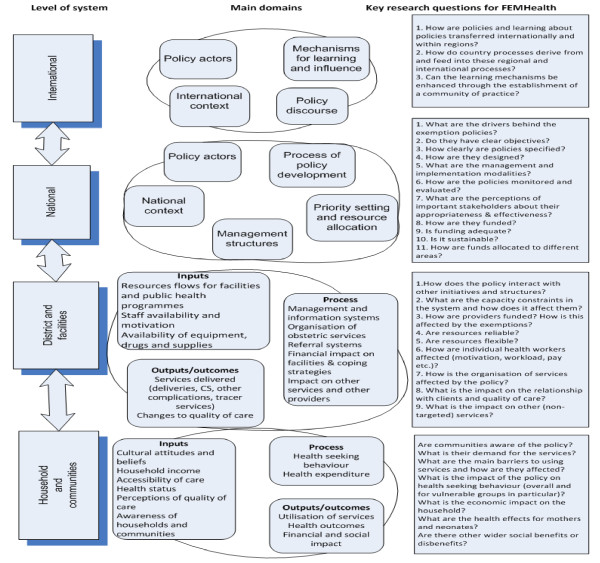
The initial conceptual framework of the FEMHealth programme.

Figure 1 presents how, during the preparatory phase, the different elements of the FEMHealth programme were conceived. It indicates the main domains of investigation and research questions for each level of the health system.

It presents the expected causal pathways at each level in the form of input-process-output-outcome configurations, but does not specifically address the linkages between the different levels nor the influence of the context. This is where the programme theory idea comes in. Although the FEMhealth programme did not set out as a theory-driven research project, we found it useful to develop a programme theory early on.

**Figure 2:**
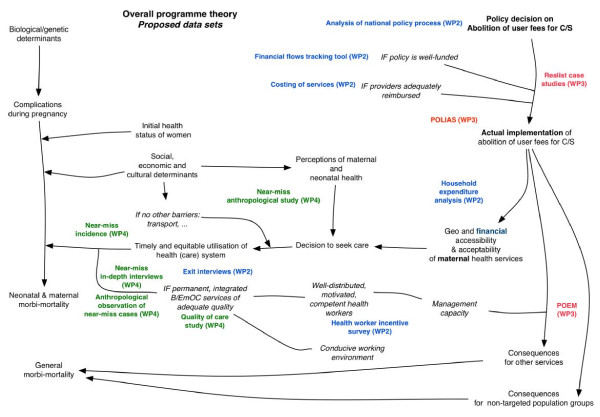
**Mapping the FEMHealth sub-studies on a simple version of the overall programme theory**^**b**^**.**

The concept of programme theory is central to theory-driven evaluation, an approach developed by Chen and Rossi [60,61]. These authors argue that for any intervention, a programme theory can be described that explains how the planners expect the intervention to reach its objective. Describing the often implicit set of assumptions that steers the choice and design of an intervention allows us to understand what is being implemented and why. It should be noted that 'theory’ is defined by Chen & Rossi as the “prosaic theories that are concerned with how human organizations work and how social problems are generated” [61]. The same can be done for a fee exemption policy. Figure 2 shows a simple version of the programme theory onto which we mapped the various sub-studies of FEMHealth. More detailed programme theories were developed to describe the effects of the policy on the local health system, to analyse the adoption and implementation of the policy by local service managers and providers, and to map how fee exemption would influence the health seeking pathways of pregnant women.

Developing a programme theory at the start of the programme served two goals. First, we aimed to facilitate a structured discussion among the researchers of their own hypotheses. As shown in Figure 1, these were developed for each level, but we felt that the work package format, favoured by the EC, posed a major risk of fragmentation. Large-scale research programmes such as FEMHealth are indeed typically organised in work packages, run by small teams of researchers, who tend to focus first on their specific research questions and only later (if time is available) on the overall objectives of the programme. Discussing the programme theory would lead, it has hoped, to better integration of the sub-studies.

In practice, the programme theory provided a framework to map the initially planned sub-studies, to find the blind spots and to better integrate the data collection. For instance, it allowed us to manage the gaps and overlaps between the work package focusing on the policy development process at national level and the team working on policy implementation within the districts. For both teams, the interface between policy/programme and implementation was important, and thinking through the transitions – from policy formulation to programme design and finally its implementation – helped us to be more efficient in data collection and in planning the analysis of data. In other cases, ideas for additional qualitative work emerged – for example, the relative absence of community-level research activities became apparent to the team - and the programme theory helped to frame this in the overall research programme. The programme theory also proved useful to review the programme’s progress and map and integrate emergent changes into the overall design. In short, it provided a common framework for seven research teams from six countries to collaborate on one overall research question. Finally, the overall programme theory was intended to provide a broad framework for cross-national comparison between the study countries. It would do so by drawing attention not only to the assessment of the actual implemented policy, but also to the specific contexts in which it took place and to the causal chains that linked the observed outcomes to the implemented policy. We discuss the main challenges we faced in the next section.

### Challenges and some possible solutions

While using concepts from complexity theory proved useful in broadening our view on fee exemption policies and in informing the overall research design, we encountered a number of challenges, only some of which were mitigated by using a programme theory perspective.

First, while the programme theory development proved useful in FEMHealth, it arguably came too late in the process. One of the strengths of using a theory-driven approach is that it demands a multi-disciplinary analysis. However, research funding mechanisms that fund such joint preparation processes during project design are rare. Indeed, most operate with tight deadlines that often preclude meetings and discussions among researchers on issues other than general outlines of a proposal. Second, most research proposal formats necessitate committing to a design and set of tools and 'deliverables’ from the very proposal development phase. This meant that in our case, the process of developing the conceptual framework and the work on the overall programme theory followed, not preceded, the specification of research tools in the proposal submitted to the funder. We found that the approved protocol allowed reasonable margins of freedom in the sense that the programme outline, the deliverables and the time table were fixed, but that the demanded level of description of the work packages left sufficient leeway to adapt the protocol to new insights and results of preliminary data analysis.

Secondly, interdisciplinary teams seem natural to research and evaluation of complex interventions, but they demand particular attention to communication and debate. Coming from the disciplines of health economics, anthropology, midwifery, statistics, demography, public health and epidemiology, the FEMHealth researchers held quite diverse sets of assumptions on how to address the policy question. Building and refining the programme theory helped to make our assumptions clear and to better take them into account in the data collection and analysis phase. However, building in enough face-to-face engagement for all team members to be comfortable with the programme theory was a challenge. In addition to disciplinary differences, we also faced the challenge of working across two languages (English and French), and being distributed across different countries.

A third challenge facing researchers working on a complex problem is the sheer volume of information that is generated if all aspects of the issue need to be covered, and thus the capacity needed to collect and process this information. A typical (human) response is to reduce complexity and to artificially limit the scope to a feasible level. This is typically done in big research projects by cutting up the issue into bits that are manageable by small research groups. During the proposal development phase of the FEMHealth project, each work package proposed a study design, with assorted methods and tools for data collection. The process of discussing the programme theory helped in reframing the protocols and data collection processes of these groups in the overall picture. As we are in the analysis phase at the time of writing, the programme theory has still to prove its usefulness in allowing integration of evidence from very diverse sub-studies. What is already clear, however, is the significant communication cost and the time required to bring together all relevant data and insights. The organisational capacity, limited project timeframe and competing demands on the time of the researchers are often major barriers to such integration.

A fourth challenge is to capture the significant relations and processes that lead to emergent behaviour in complex systems, or in the case of a policy implementation, the responses that result from the interaction between key actors and institutions in terms of structure and culture. To the extent that some of these responses are emergent and thus not predictable, total planning for data collection plan is not possible, and flexibility needs to be built in. In FEMHealth, the qualitative data collection moments proved most useful to explore such emergent issues. These included, for example, policy ethnographies and interviews with key actors to document the interactions at the global-national interface and to describe the national-level policymaking processes. The policy implementation process was documented by a combination of methods, including interviews of actors at different levels of the health system. Another approach was to try and document outcome patterns and to explore the unexpected results through mixed research methods. To this end, we set out to assess the effects of the policy by a combination of measuring changing near-miss incidence, conducting observations in facilities, exit interviews and in-depth interviews with patients. Yet another strategy to capture emergence was to use realist evaluation as the approach to the study of the policy adoption by health service managers and providers. A programme theory was developed on the basis of a literature review and tested in two sites in three of the countries. This sub-study reduced the complexity challenge by zooming in on a specific aspect of the policy implementation process, while at the same time allowing for a complexity perspective in the analysis of that aspect.

Finally, if flexible designs are required, the question of replicability pops up. Whereas replicability of other kinds of studies rely mainly on the quality of the study protocol and the adherence to it, studies of complex issues that have an important emerging part need to ensure traceability of changes made to the protocol and to clearly document why changes were made in the first place.

In studies dealing with complex issues, the researchers need to adopt an adaptive attitude during the data collection to keep in tune with the evolving understanding of the issue under study. They need to be able to identify and capture unforeseen events that may be critical for the study. In other words, analytical capacity and research experience matters, as data collection through closed questionnaires and quantitative surveys will not be sufficiently flexible.

The programme theory may also prove helpful in this respect. Yin advocates the use of multiple cases in a replication process to enhance the theory-building capacity of this design: “*The remedy is to consider a case study, as a unit, to be equivalent to an experiment, as a unit; multiple-case studies may then be considered equivalent to multiple experiments*” [62]. Yin argues that replication logic can be based on the theory behind the cases. In order to test this hypothesis, 'critical’ cases are selected and their results compared on the basis of the initial hypothesis. If the same results are found and rival hypotheses can be eliminated, the theory is strengthened. Through this process of analytical generalisation, findings of case studies can thus be generalised to the theoretical propositions (not to populations, as quasi-experimental methods attempt to do) [63]. This is in line with the principles of theory-based evaluation [64,65].

A last challenge in the case of FEMHealth is to go beyond sub-study specific analysis and cut across databases to do a comprehensive analysis at country-level, and then at cross-national level. One solution is to dissolve work package groups and regroup researchers in country-specific teams that focus on integrative analysis of the cases. This is the stage in which the project finds itself at the writing of this paper, with limited time to undertake this most interesting part of the analysis. A common solution is for researchers to continue to invest after project funding has ceased. This, however, demands a degree of institutional support and capacity, which is most challenging in under-funded research institutions in many low-income countries.

## Conclusions

Much of the conceptual confusion surrounding complex interventions can be eliminated by differentiating between simple, complicated and complex interventions, and by assessing the knowledge base, the degree of consensus and the implementation capability. This paper examined how fee exemption policies, like many health policy changes, fit the criteria for complexity, and what the implications are for evaluation design. It has presented some practical experiences of research design to meet the challenges of complexity, but also some outstanding issues.

To generate evidence and learning in complex situations or regarding complex interventions, research and evaluation methods need to be able to deal with the key issues of loosely-coupled networks of actors that make up the health system (and thus non-linear relations, time delays in feedback loops, self-organisation and emergence of new behaviour), and the embeddedness of health systems in multi-layered contexts and systems. Research and evaluation approaches therefore need not only to provide a holistic and systemic view on the problem and/or solution. Designs also need to be considered as dynamic, so that they can be adapted in response to the insights that emerge.

Thinking about the programme theory underlying the fee exemption policies helped not only to bring together FEMHealth researchers working on different aspects and tools, but also to see the gaps and overlaps in the planned research activities. It also helped to identify potential unknown issues and will structure the country-level analysis as well as the cross-national comparisons. Whether the programme theory can fulfil its claimed potential to help make sense of the findings is yet to be tested. Experience from other studies allows for some moderate optimism. However, the biggest challenge complexity throws at health system researchers is to deal with the unknown unknowns and the consequence that complex issues can only be understood in retrospect. From a complexity theory point of view, only plausible explanations can be developed, not predictive theories, and here theory-driven approaches may help.

## Endnotes

^a^See http://www.abdn.ac.uk/femhealth for background on the project. The project runs from 2011 to 2013, and includes partners from the UK (University of Aberdeen and the London School of Hygiene and Tropical Medicine), from Belgium (the Institute of Tropical Medicine), Burkina Faso (AfricSanté), Benin (CERRHUD), Mali (MARIKANI), and Morocco (the National School of Public Health).

^b^The different colours represent different work packages within the research programme – WP2 focused on health policy and financing issues, WP3 on local health system issues, and WP4 on quality of care and utilisation responses.

## Competing interests

The authors declare that they have no competing interests.

## Authors’ contributions

BM contributed to the conception of this manuscript, the section on complexity, and the design of the FEMHealth programme. SVB contributed to the conception of this manuscript and the section on complexity. VDB contributed to the section on complexity and the design of the FEMHealth programme. SW contributed to the conception of this manuscript, the section on complexity, and the design of the FEMHealth programme. All authors read and approved the final manuscript.

## Pre-publication history

The pre-publication history for this paper can be accessed here:

http://www.biomedcentral.com/1472-6963/13/469/prepub
